# Validation of student academic advising and counseling evaluation tool among undergraduate nursing students

**DOI:** 10.1186/s12909-023-04115-5

**Published:** 2023-03-02

**Authors:** Abeer Selim, Abeer Omar, Shaimaa Awad, Eman Miligi, Nahed Ayoub

**Affiliations:** 1 Faculty of Nursing, Psychiatric and Mental Health Nursing Department, Mansoura University, Mansoura, Egypt; 2 College of Nursing, King Saud Bin Abdulaziz University for Health Sciences, Riyadh,3105, P.O. Box 3660, Riyadh, 11481 Saudi Arabia; 3 King Abdullah International Medical Research Center, Riyadh, Saudi Arabia; 4 Trent/Fleming School of Nursing, Trent University, 1600 West Bank Drive, Peterborough, ON K9L 0G2 Canada; 5 Faculty of Nursing, Nursing Administration Department, Cairo University, Cairo, Egypt; 6 Faculty of Nursing, Gerontological Nursing Department, Mansoura University, Mansoura, Egypt; 7 College of Nursing, Umm Al Qura University, Makkah, Saudi Arabia

**Keywords:** Academic advising, Academic counseling, Nursing students, Evaluation tool

## Abstract

**Background:**

Academic advising and counseling services support students in achieving their educational outcomes. Unfortunately, there is a paucity of research on academic advising and student-support systems among nursing students. Therefore, the current study aims to develop a student academic advising and counseling survey (SAACS) and measure its validity and reliability.

**Methods:**

Cross-sectional design was used to collect online self-administered data from undergraduate nursing students in Egypt and Saudi Arabia. The SAACS is developed based on relevant literature and tested for content and construct validity.

**Results:**

A total of 1,134 students from both sites completed the questionnaire. Students’ mean age was 20.3 ± 1.4, and the majority of them were female (81.9%), single (95.6%), and unemployed (92.3%). The content validity index (CVI) of SAACS overall score (S-CVI) is 0.989, and S-CVI/UA (universal agreement) is 0.944, which indicates excellent content validity. The overall SAACS reliability showed an excellent internal consistency with a Cronbach’s Alpha of 0.97 (95% CI: 0.966 – 0.972).

**Conclusions:**

The SAACS is a valid and reliable tool for assessing students’ experience with academic advising and counseling services and can be utilized to improve those services in nursing school settings.

**Supplementary Information:**

The online version contains supplementary material available at 10.1186/s12909-023-04115-5.

## Background

University students experience a high stress level, which highlights the need for adequate advising and support services to enable students to achieve their goals [1]. Stress triggers several mental health problems such as depression, anxiety, sleep disorders, eating disorders, alcohol, and substance use among university students [2–7]. Evidence showed that university students, particularly those in health science departments, have higher rates of anxiety and depression than the general population. [8, 9], along with a higher rate of academic stress, mainly among nursing and medical students [10–12]. Nursing students face many academic stressors, particularly during their clinical training. Other stressors are related to time management in balancing academic and social commitments [13, 14]. However, the most common stressors facing nursing students are related to clinical training and workload [15–18].

Student academic advising and counseling services significantly support students, particularly those who experience mental health issues, to achieve their educational outcomes [1, 19]. However, there are distinctions between academic advising and counseling services [20]. Academic Advising services offer students with academic, transfer, and career support. While counseling services help students who have psychosocial or mental health concerns via assessment, intervention, and/or referral [16, 21]. However, a large population of students is frequently unscreened and unattended when counseling and treatment are needed [9]. In addition, the COVID-19 pandemic has disrupted nursing education and forced the transition to the remote and online modes of teaching for theory and clinical courses [22]. COVID-19 pandemic disruptive challenges have greatly impacted nursing students’ mental health, mounting depression, anxiety, fear, stress, and sleep disturbances [23]. Which, in turn, heightened the need for mental health services and academic advising. However, the pandemic has affected academic advising delivery and moved it to remote sessions [24].

Improving nursing students' academic and learning experiences is vital in enhancing their learning process, academic achievement, and mental well-being [25]. In Arab countries, most students graduate from public high schools with Arabic-based instruction systems making the transition to an English-based instruction system at the college level challenging and indicating the need for a tailored student-support approach. There is a paucity of research on academic advising and student-support systems among university students in Arab countries [26]. Additionally, there is a need for a robust assessment that includes university students' feedback and evaluation of the advising and counseling service to identify areas for improvement [27]. To our knowledge, previous studies at data collection sites did not establish the validity and reliability of the tools used in evaluating academic advising.

## Methods

### Aim

The current study aimed to develop a student's academic advising and counseling tool in nursing schools and measure the developed tool’s validity and reliability.

The lack of evaluation of academic advising and counseling services drives the need to develop a reliable and valid evaluation tool. Thus, the current study question is “What is the validity and reliability of the student academic advising and counseling survey?”.

### Design

A cross-sectional design was used to conduct the current study and measure the developed tool’s validity and reliability. In addition, the study investigators used the quantitative approach of measuring the psychometric properties of the developed tool.

### Sample

Study participants included undergraduate nursing students from the first to the fourth year who were willing to participate and gave informed consent. Exclusion criteria included postgraduate students and those who refused to participate in the study. There is a debate in the literature about the appropriate sample size for factor analysis [28]. The sample size might be determined based on the ratio of respondents to items on the scale, with at least 10 participants for each scale item. Other researchers have recommended a 200–300 sample size appropriate for factor analysis. Others have suggested a minimum of 300–450 [29, 30]. Comrey and Lee (1992) rate sample size for scale development as; 100 = poor, 200 = fair, 300 = good, 500 = very good, ≥ 1,000 = excellent [31]. According to Boateng and colleagues (2018), a sample size equal to or over 1,000 is excellent for establishing construct validity. In addition, a larger sample size indicates lesser measurement errors and more stable factor loadings, replicable factors, and generalizable results to the true population structure [32]. Therefore, the study investigators decided to recruit more than 1,000 participants for the current study. The total number of undergraduate nursing students at both sites is 3,524 (2,974 in Egypt and 550 in Saudi Arabia). A convenience sampling method was used to recruit 1,134 students who completed the survey (729 from Egypt and 405 from Saudi Arabia). This sample size demonstrates a response rate of 32.2%.

### Setting

Data collection took place at two nursing schools; the faculty of nursing in Egypt and the college of nursing in Saudi Arabia. Academic advising at both sites is focused on guiding students to achieve their academic goals through monitoring their academic performance, attendance, and professional conduct. It also helps refer students to supportive services, such as psychological, social, and/or any other health services. However, one difference between the two sites is academic registration. In Egypt, academic advisors and academic affairs staff assist students with their course registration and academic schedules to avoid courses overlapping. While in the Saudi school of nursing, this service is handled by academic affairs staff, not advisors.

### Study instrument

An online self-administered survey was used to collect data from nursing students about socio-demographic information and student academic advising and counseling evaluation. Socio-demographic data questions included the profile of students’ age, gender, academic level/year, marital status, number of children, residence, GPA, employment, leisure activities, physical and mental problems/disorders, frequency of individual meetings with the adviser, and preferred way of communication with academic advisors. The study investigators tried to adhere to Boateng et al. (2018) phases for scale development (item development, scale development, and scale evaluation**)** as well as Watkins's (2018) exploratory factor analysis.

### Phase 1: item development

#### Step 1: Identification of domain and Item generation

After reviewing relevant literature [1, 33–35], we developed the student academic advising and counseling survey (SAACS) with the main purpose of assessing the students’ satisfaction with their academic and counseling provided services using a valid and reliable tool. The initial generated survey pool comprised 28 items. Each item rated on a five-point scale scored as follows: strongly agree = 5; agree = 4; neither agree nor disagree = 3 disagree = 2; strongly disagree = 1.

#### Step 2: Content validity: assessing if the items adequately measure the domain of interest

The researchers followed Polit and colleagues (2007) recommendations to establish content validity [36]. For that reason, we sent the survey to five experts in the field of student academic advising and counseling along with a rating scale to evaluate the survey items' relevance on a Likert scale from 1 to 4. Ratings of 3 or 4 indicate item relevance, while 1 and 2 ratings designate non-relevance of the items. The invitation sent to the experts included clear instructions about rating each survey item and recommending any modification, deletion, or addition of items. We also requested that the panel provide a second round of review after considering their recommendations. Experts’ feedback included a rating of each item and recommended a few modifications of some statements, deletion of 12 items as they were redundant, and adding three items. The content validity index was computed for each item (CVI), and the overall scale (S-CVI) as well as S-CVI/UA (universal agreement, when experts give a rating of 3 or 4 to the items). The I-CVI ranged from 0 to 1 with a total panel agreement of 15, and the S-CVI = 0.643. Items that ≥ 0.78 were kept (15 items), the remaining items were deleted, and three additional items were added as recommended by the panel (see Additional file 1:  appendix 1).

Accordingly, the final version of the survey had 18 items, and we sent it back to the same panel for the second round of content validity. I-CVI of the final version of SAACS ranged from 0.8 to 1 with a total agreement of 17, S-CVI = 0.989, and S-CVI / UA = 0.944 indicating excellent content validity (see Additional file 1: appendix 2). Survey items from 1 to 17 are rated on a five-point scale scored as follows: strongly agree = 5; Agree = 4; neither agree nor disagree = 3 disagree = 2; strongly disagree = 1, and item 18 is rated as strongly satisfied = 5; satisfied = 4; neither satisfied nor dissatisfied = 3 dissatisfied = 2; strongly dissatisfied = 1.

### Phase 2: Scale development

#### Step 3: Pre-testing questions: ensuring the questions and answers are meaningful.

Further, we piloted the final SAACS on ten students who confirmed the clarity of the survey with no further modification.

#### Step 4: Survey administration and sample size: gathering enough data from the right people

See the sample section above.

#### Step 5: Item reduction

See the results section; testing data factorability below (Inter-item and Item-total correlations). Our sample did not suffer from incomplete cases as all items had to be answered by the respondents online.

#### Step 6: Extraction of factors

For factor retention, we used Kaiser or mineigen greater than 1 criterion, the percentage of variance, the scree test/plot, the parallel analysis, and the minimum average partial (for more details, see the statistical analysis section below and scree plot in results section). We tested all possible factor solutions: one factor-, two factors-, and three factors-solutions; in all solutions, we found all items saliently loaded on the same factor (factor loadings ranged from 0.7 to 0.9) without any cross-loadings. This assured us of the adequacy of the one factor-solution.

### Phase 3: Scale evaluation

Furthermore, the researchers have established the construct validity of the developed tool using exploratory factor analysis. The internal consistency of the tool was measured to ensure reliability by calculating Cronbach Alpha.

### Data collection

The researchers commenced data collection using an online survey at two schools of nursing in Egypt and Saudi Arabia after securing the Institutional Review Board (IRB) at each site. Those two sites were selected conveniently because of the collaboration between the research teams in both settings and the feasibility of data collection from two different Arabic-speaking countries. At the time of data collection, COVID -19 restrictions were reduced due to relative control via personal protective measures, social distance, and immunization. Hence, students at the two sites spent their clinical training at the college nursing labs and hospitals. Only theory classes and academic advising sessions were delivered online.

The survey link was sent to all undergraduate nursing students in the BSN program via emails and social communication applications (WhatsApp groups). Each student batch in each academic year has a WhatsApp group in both countries. Weekly reminders were sent via emails to enhance data collection. Data collection steps were standardized at both sites using an online survey, students' emails, and sending weekly reminders for the whole undergraduate students. Data collection took two months at each site, from March to April 2021.

### Statistical analysis

Data entry, cleaning, and analysis were performed using the R statistical package, version 4.1.3 (R Core Team; 2020). Continuous data were summarized using means and standard deviations. Student t-test or Welch t-test (in case of unequal variances) were used to comparing continuous variables between groups; despite the ordinal nature of the 5-point Likert scale items, we used t-test to compare them between groups as it was found to outperform the nonparametric methods in comparing discrete numeric variables [37] and simulation studies proved that they have the same power [38]. The categorical variables were summarized using frequencies and percentages. The Chi-square test or Fisher’s exact tests were used to compare categorical variables as appropriate.

The psych package (Version 2.2.5) was used to conduct the exploratory factor analysis. Bartlett's test of sphericity [39] was used to ensure that the correlation matrix was not an identity matrix. The sample adequacy was tested by the Kaiser–Meyer–Olkin (KMO) test where the KMO statistic was required to be above a minimum of 0.50 [40]. We used a polychoric correlation matrix to account for the ordinal nature of the 18 items. After confirming its factorability, we submitted it for EFA. Since we aimed to identify the latent constructs of the 18-item academic advising services survey, we used common factor analysis. Due to the ordinal nature of the 18 items, we used an iterated principal axis factoring for factor extraction, which is the preferred extraction method for this type of scale [28].

We used five methods for factor retention: Kaiser or mineigen greater than 1 criterion (factors with eigenvalues above 1) [41, 42], the percentage of variance (the solutions that account for at least 60%), the scree test/plot (the number of factor points above the break to be retained and excluding the breakpoint) [43], the parallel analysis and the minimum average partial. The construct was developed, assuming its factors would be correlated. Therefore, we used a direct oblimin rotation, which is the preferred method for oblique rotation [28].

## Results

### Students’ characteristics

The baseline characteristics of participants are summarized in Table 1. A total of 1,134 students (205 males and 929 females) completed the survey with an average completion time of 7.3 ± 19.1 min. Students’ mean age was 20.3 ± 1.4, and the majority of them were single (95.6%), living with their families (93.8%), and unemployed (92.3%). The mean GPA was 3.5 ± 0.6. More than a quarter of the nursing students (28.7%) reported mental illness, and the most frequent complaint was insomnia (18.3%). Headache was the most frequently reported physical complaint (20.4%). Also, more than a third of the participating students did not have any meetings with their advisors (36.5%), more than half of students preferred social media as a means of communication with their advisors (51.6%), and the majority of them were satisfied with their academic advising (74.2% had a mean score of more than 3).

**Table 1:** Students’ baseline characteristics by country of residence

Variables	Total (*n* = 1134)	
	*n*	*%*
Age in years (mean ± SD)	20.3 ± 1.4	
Country		
Egypt	729	64.29
Saudi Arabia	405	35.71
Gender		
Male	205	18.1
Female	929	81.9
Academic year		
First	275	24.3
Second	308	27.2
Third	296	26.1
Fourth	255	22.5
Marital status		
Single	1084	95.6
Married	49	4.3
Divorced	1	0.1
Children (yes)	46	4.1
Residence		
Alone	21	1.9
With families or relatives	1064	93.8
With classroom mates	49	4.3
Current GPA(mean ± SD)^a^	3.5 ± 0.6	
Employment		
Unemployed	1047	92.3
Part-time	78	6.9
Full-time	9	0.8
Frequency of leisure activities per week		
None	224	21.5
Once/rarely	284	25.0
Sometimes	413	36.4
Often	142	12.5
Always	51	4.5
Self-reported mental disorders	326	28.7
Insomnia	207	18.3
Depression	117	10.3
Mood disorders	16	1.4
Anxiety	77	6.8
Eating disorders	85	7.5
Suicidal thoughts	26	2.3
Self-harm behaviors	21	1.9
Obsessive–compulsive disorders	26	2.3
Self-reported physical disorders	432	38.1
Anemia	230	20.3
Headache	231	20.4
Gastrointestinal disease	93	8.2
Cardiovascular disease	13	1.1
Respiratory disease	37	3.3
Endocrine disorder	20	1.8
Frequency of individual meetings with the adviser		
None	414	36.5
Once/rarely	344	30.3
Sometimes	246	21.7
Often	36.5	36.5
Always	61	5.4
Preferred way of communication with the adviser		
Face to face	541	47.7
Email	101	8.9
Phone	141	12.4
Text messaging	83	7.3
Social media	585	51.6
Satisfaction with academic advising		
Yes	841	74.2
No	293	25.8

^a^GPA was missing for 71 respondents

### Validity and reliability of SAACS

#### Testing data factorability

**Fig. 1:**
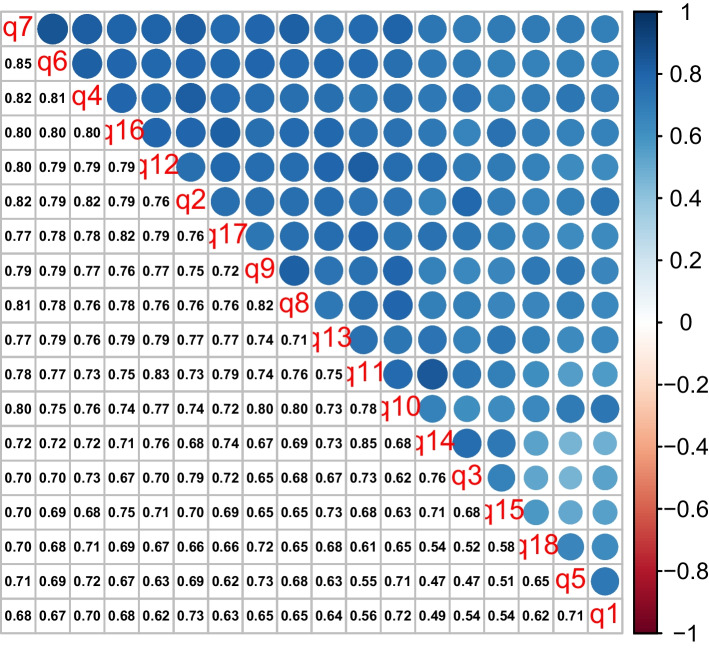
SAACS EFA Polychoric Correlation Matrix

Exploratory factor analysis was conducted to determine the validity and reliability of SAACS. Although the skewness and kurtosis of all 18 items were not extreme, we found that Mardia’s multivariate skewness and kurtosis were both statistically significant (*p* < 0.001). This deviation from normality is attributed to the ordinal nature of the 5-point Likert scaled items; therefore, we used a polychoric correlation matrix (see Fig. 1) for the input for our exploratory factor analysis. All inter-item correlations were found above 0.3 by far.

The results of Bartlett’s test of sphericity concluded that the correlation matrix was not random (χ2(153) = 24,667.75, *p* < 0.001), and the sample adequacy was confirmed by the Kaiser–Meyer–Olkin test (KMO), which was well above the minimum standard for conducting factor analysis (KMO statistic = 0.97). Therefore, we were assured about the factorability of the correlation matrix. We computed the adjusted item-total correlations; they ranged from 0.67 to 0.86, providing more evidence about how well each item is correlated to the sum of the rest of the items, excluding itself.

#### Factor retention methods

**Fig. 2:**
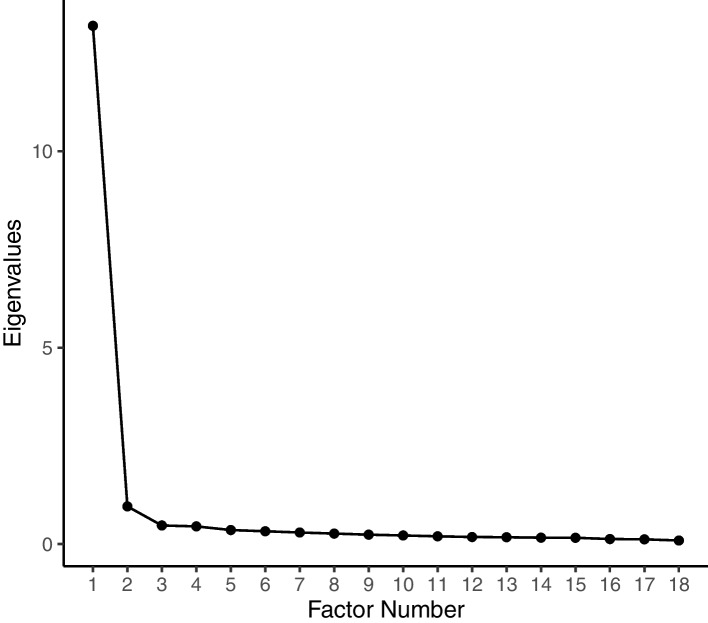
SAACS EFA Scree Plot

We examined different methods for factor retention that showed a different number of factors required. The initial Eigenvalues that resulted from the implemented principal axis factoring are presented in Additional file 1: appendix 1 and visualized by the scree plot (Fig. 2).

Both of the two methods pointed prominently that only one factor is required; only one factor had an Eigenvalue above one (13.19) and accounted for 73% of the total variance alone. It was evident from the scree plot that there is only one factor above the breakpoint. However, the parallel analysis and the minimum average partials methods indicated that three and two factors are required, respectively. Upon examining the one, two, and three-factor solutions, we found all the 18 items saliently loaded on one factor more than the others (Additional file 1: Appendix 3). Consequently, we were assured of the adequacy of the one-factor solution, which is presented together with the descriptive statistics of the 18 items in Table 2. The overall reliability testing showed an excellent internal consistency with a Cronbach’s Alpha of 0.969 (95% CI: 0.966 – 0.972).

**Table 2:** Descriptive Statistics and Factor Loadings of Student Academic Advising and Counseling Survey (SAACS) EFA

SAACS item	Mean	SD	Skewness	Kurtosis	Factor loadings	*h*^*2*^
1. My advisor is knowledgeable about the university, college`s policies, and procedures	3.81	0.88	-0.94	1.4	**0.6**	0.6
2. I feel comfortable speaking with my advisor about academic matters	3.57	0.98	-0.79	0.45	**0.8**	0.8
3. I feel comfortable speaking with my advisor about a personal matter	3.18	1.11	-0.26	-0.65	**0.6**	0.6
4. My advisor understands my academic development needs	3.58	0.96	-0.73	0.45	**0.8**	0.8
5. My advisor treats me with respect	4.01	0.83	-0.93	1.44	**0.6**	0.6
6. My advisor helps me set my academic plan and to anticipate opportunities promptly	3.57	1	-0.63	0.08	**0.8**	0.8
7. My advisor processes my requests on time	3.58	0.95	-0.62	0.24	**0.8**	0.8
8. I can meet with my advisor in a reasonable amount of time	3.54	0.96	-0.6	0.19	**0.8**	0.8
9. I am satisfied with the communication methods with my advisor	3.61	0.01	-0.74	0.23	**0.8**	0.8
10. My advisor is knowledgeable about what applies to my major	3.6	0.97	-0.66	0.21	**0.7**	0.7
SAACS item	Mean	SD	Skewness	Kurtosis	Factor loadings	*h*^*2*^
11. My advisor gives attention to my personal and social development	3.23	1.08	-0.23	-0.59	**0.8**	0.8
12. My advisor evaluates my progress in meeting my graduation requirements	3.44	1	-0.55	-0.03	**0.8**	0.8
13. My advisor has positively impacted my continued enrollment in the nursing program	3.51	1.01	-0.63	0.09	**0.8**	0.8
14. My advisor is sensitive to my psychological changes	3.09	1.11	-0.18	-0.72	**0.7**	0.7
15. I prefer to contact my advisor when there is a stressful situation	3.38	1.11	-0.55	-0.38	**0.6**	0.6
16. I would recommend my advisor to other students	3.61	0.98	-0.67	0.33	**0.8**	0.8
17. My advisor is a source of social support	3.54	1	-0.65	0.18	**0.8**	0.8
18. How would you rate the overall effectiveness of your advising process?	3.68	0.98	-0.58	0.23	**0.6**	0.6
SS loadings	12.9					
Percentage of variance	72%					
Internal consistency	Cronbach's Alpha = 0.969 (95% CI: 0.966 – 0.972)					

*h*^*2*^ = commonalities. Factor loadings above .40 are in bold. *SS* sum of squares

## Discussion

Academic advising is an integral aspect of university life [44]. Student academic advising and counseling is a supporting and empowering process that enables students to achieve their academic and personal goals during their learning journey. Academic advising and counseling services advocate for students’ rights and ease their academic paths.

In the current study, the majority of participating nursing students were females, single, living with their families and unemployed. The students’ mean GPA was satisfactory. Apparently, nursing students preferred social media to communicate with their advisors and were satisfied with their academic advising. Students tend to value the support of their academic advisors [44]. In that sense, Gordon-Starks has defined academic advising as relationship building in which academic advisors play a pivotal role in influencing their advisees’ lives while guiding and mentoring them during their academic journey [45]. This might explain the students’ satisfaction with their academic advising. However, more than a third of them did not meet with their advisors.

Academic advisors’ knowledge and skills significantly influence satisfaction with academic advising. Likewise, the research highlighted the influence of advisors’ knowledge and skills on students’ satisfaction with their academic advising [46]. In this regard, one of the common recommendations to improve academic advising is increasing training opportunities for advisors [47, 48]. Hamlet (2017) also reported that effective academic advising needs time and knowledge [49]. Furthermore, a Jordanian study reported that students demanded frequent meetings with their advisors to improve their academic advising [47]. The research also found a significant positive correlation between students’ GPA and their satisfaction with academic advising [50]. Further correlates and predictors of satisfaction with academic advising need further studies with correlational designs.

In the current study, insomnia and headache were the most frequently reported mental health issues. These findings are in the same direction as Correa et al. (2021). The only difference is Correa and colleagues (2021) [51] found that insomnia was a result of severe headaches, which is not clear in the current study. Compared to the general population and other university students, nursing students are suffering from higher rates of mental problems such as anxiety and depression [8, 9] due to exposure to higher levels of academic stress [10–12]. In addition, clinical training and time management are the common significant stressors confronting nursing students [13, 14]. Therefore, academic advising is essential in supporting, mentoring and referring those students to appropriate mental health services.

The current study investigators examined the validity and reliability of the evaluation survey to ensure a comprehensive evaluation of the academic advising process. Before data collection, we evaluated the survey content validity of the items and the scale following Polit et al. (2007) [36] to develop a tool and have two rounds of expert evaluation and rating as well as the acceptable values based on the number of panel experts. Initially, we developed the tool items based on a thorough literature review of academic advising studies and tools. The initial draft of the tool (28 items) were sent to a panel of 5 experts with a relevance rating scale. The panel ratings were used to calcite the initial CVI and their recommendations regarding; retention, revision, deletion and addition of items were considered in revisiting the tool. The second version of the tool was developed and sent back to the same panel to be re rated after the modifications and hence, the CVI was computed for the final version of the tool (18 items) that exhibited strong content validity and we used it for data collection.

The construct validity of SAACS was established via EFA. Using evidence- based EFA, we followed Watkins’s (2018) best practice guide in selecting the software, and EFA decisions such as number of participants, distributional properties of the data, model of factor analysis, estimation method, the number of retained factors, rotation of factors, and interpretation and reporting of results (see data management and analysis, and results sections for more details) [28].

The EFA results preferred a single construct/factor solution related to academic advising and counseling evaluation as evidenced by the scree plot (see Fig. 1), factor loadings, and percentage of variance. Also, conceptually, all of the survey items evaluated students’ satisfaction with the academic advising process, and eventually supported one-factor scale.

### Strengths and limitations

The current study's purpose was to measure the validity and reliability of academic advising and counseling evaluation tool (SAACS) for nursing students. The SAACS has proved to be an excellent valid, and reliable tool for assessing students’ experience with academic advising and counseling services at the university level. In addition, the relatively large sample size was one of the study's strengths. However, the current study utilized a quantitative cross-sectional design, and no qualitative arm was used to understand the experience of academic advising and counseling in nursing schools. An additional limitation is that the results of this study are generalized only to the two data collection settings. Also, the current study did not measure the concurrent validity of the survey due to lack of current valid tools in the region.

### Implications for practice

Students need academic advising to achieve their academic goals in a supportive environment. In response to the gap in the literature on valid and reliable academic advising evaluation tools, we have developed a valid and reliable tool. We believe this tool will help evaluate and improve nursing schools' academic advising and counseling process. In addition, using this tool in our nursing sites can help academic advisors address the gaps in the services provided and modify the delivery way according to students' preferences.

### Conclusion and recommendations

The Student Academic Advising and Counseling Survey (SAACS) is a valid and reliable tool for assessing students’ experience with academic advising and counseling services at the university level. This highly reliable tool can improve academic advising and counseling services in nursing school settings. The study investigators recommend conducting confirmatory factor analysis using the developed tool in another country. We also recommend utilizing a mixed-method approach to examine the students’ evaluation and perception of the academic advising and counseling services in their settings. Teasley and Buchanan et al. (2013) argued that evaluating academic advising using surveys paired with qualitative methods such as structured interviews will give a well-rounded view [52].

## Supplementary Information

**Additional file 1.**

## Data Availability

The datasets generated and/or analyzed during the current study are not publicly available but are available from the corresponding author on reasonable request.
