# Supplementary material for: Validation of student academic advising and counseling evaluation tool among undergraduate nursing students
Source: BMC Med Educ. 2023 Mar 2;23:139. doi: 10.1186/s12909-023-04115-5 (PMC9978278; doi:10.1186/s12909-023-04115-5)
Supplement: Supplementary file 1 — Additional file 1. [file 12909_2023_4115_MOESM1_ESM.docx]

Appendix 1

*First Round Expert Panel rating of Student Academic Advising and Counseling Survey Initial Version*

| Initial items (28) | Rater 1 | Rater 2 | Rater 3 | Rater 4 | Rater 5 | Experts in Agreement | Item CVI |
| --- | --- | --- | --- | --- | --- | --- | --- |
| 1 | 4 | 4 | 4 | 4 | 4 | **5** | **1** |
| 2 | 4 | 3 | 4 | 4 | 4 | **5** | **1** |
| 3 | 4 | 4 | 4 | 4 | 4 | **5** | **1** |
| 4 | 4 | 4 | 4 | 4 | 4 | **5** | **1** |
| 5 | 1 | 4 | 1 | 2 | 3 | 2 | 0.4 |
| 6 | 1 | 2 | 1 | 1 | 2 | 0 | 0 |
| 7 | 4 | 4 | 4 | 4 | 3 | **5** | **1** |
| 8 | 4 | 4 | 4 | 4 | 4 | **5** | **1** |
| 9 | 1 | 4 | 1 | 1 | 4 | 2 | 0.4 |
| 10 | 4 | 4 | 4 | 4 | 4 | **5** | **1** |
| 11 | 4 | 4 | 4 | 4 | 4 | **5** | **1** |
| 12 | 1 | 4 | 1 | 4 | 1 | 2 | 0.4 |
| 13 | 2 | 4 | 2 | 1 | 1 | 1 | 0.2 |
| 14 | 1 | 3 | 2 | 1 | 1 | 1 | 0.2 |
| 15 | 3 | 3 | 4 | 4 | 4 | **5** | **1** |
| 16 | 1 | 3 | 1 | 1 | 1 | 1 | 0.2 |
| 17 | 1 | 2 | 1 | 4 | 1 | 1 | 0.2 |
| 18 | 2 | 3 | 1 | 1 | 1 | 1 | 0.2 |
| 19 | 3 | 3 | 3 | 4 | 4 | **5** | **1** |
| 20 | 4 | 4 | 4 | 4 | 4 | **5** | **1** |
| 21 | 2 | 1 | 1 | 1 | 2 | 0 | 0 |
| 22 | 2 | 3 | 2 | 4 | 1 | 2 | 0.4 |

Excellent agreement and ICV are in bold.

Appendix 1

*First Round Expert Panel rating of Student Academic Advising and Counseling Survey Initial Version (Cont’d)*

| Initial items (28) | Rater 1 | Rater 2 | Rater 3 | Rater 4 | Rater 5 | Experts in Agreement | Item CVI |
| --- | --- | --- | --- | --- | --- | --- | --- |
| 23 | 4 | 3 | 3 | 4 | 4 | **5** | **1** |
| 24 | 4 | 3 | 3 | 4 | 4 | **5** | **1** |
| 25 | 3 | 4 | 4 | 3 | 4 | **5** | **1** |
| 26 | 1 | 1 | 2 | 1 | 1 | 0 | 0 |
| 27 | 1 | 4 | 3 | 1 | 1 | 2 | 0.4 |
| 28 | 4 | 4 | 4 | 3 | 3 | **5** | **1** |
| S-CVI | 0.643 | | | | | | |
| Total Agreement | 15 | | | | | | |
| S-CVI / UA | 0.536 | | | | | | |

Excellent agreement and ICV are in bold.

Appendix 2

*Second Round of Expert Panel rating of Student Academic Advising and Counseling Survey* (*SAACS) Final Version*

| SAACS items (18) | Rater 1 | Rater 2 | Rater 3 | Rater 4 | Rater 5 | Experts in Agreement | Item CVI |
| --- | --- | --- | --- | --- | --- | --- | --- |
| 1 | 4 | 4 | 4 | 4 | 4 | **5** | **1** |
| 2 | 4 | 4 | 4 | 4 | 4 | **5** | **1** |
| 3 | 4 | 4 | 4 | 4 | 4 | **5** | **1** |
| 4 | 4 | 4 | 4 | 4 | 4 | **5** | **1** |
| 5 | 4 | 4 | 4 | 4 | 4 | **5** | **1** |
| 6 | 4 | 4 | 4 | 4 | 4 | **5** | **1** |
| 7 | 4 | 4 | 4 | 4 | 4 | **5** | **1** |
| 8 | 4 | 4 | 4 | 4 | 4 | **5** | **1** |
| 9 | 4 | 4 | 4 | 4 | 4 | **5** | **1** |
| 10 | 4 | 4 | 4 | 3 | 4 | **5** | **1** |
| 11 | 4 | 4 | 4 | 4 | 4 | **5** | **1** |
| 12 | 4 | 3 | 4 | 4 | 4 | **5** | **1** |
| 13 | 4 | 4 | 4 | 4 | 4 | **5** | **1** |
| 14 | 4 | 4 | 4 | 4 | 4 | **5** | **1** |
| 15 | 4 | 4 | 4 | 3 | 3 | **5** | **1** |
| 16 | 4 | 3 | 4 | 4 | 4 | **5** | **1** |
| 17 | 4 | 4 | 4 | 4 | 3 | **5** | **1** |
| 18 | 4 | 3 | 4 | 4 | 2 | **4** | **0.8** |
| S-CVI | 0.989 | | | | | | |
| Total Agreement | 17 | | | | | | |
| S-CVI / UA | 0.944 | | | | | | |

Excellent agreement and ICV are in bold.

Appendix 3

*Summary of Student Academic Advising and Counseling Questionnaire EFA for the One, Two, and Three-Factors Solutions*

| SASCQ item | One Factor solution | Two Factor solution | | Three Factor solution | | |
| --- | --- | --- | --- | --- | --- | --- |
|  | F1 | F1 | F2 | F1 | F2 | F3 |
| 1. My advisor is knowledgeable about the university, college`s policies, and procedures. | **0.6** | **0.7** | 0.3 | **0.8** | -0.3 | -0.1 |
| 2. I feel comfortable speaking with my advisor about academic matters. | **0.8** | **0.9** | 0.1 | **0.9** | -0.2 | 0.2 |
| 3. I feel comfortable speaking with my advisor about a personal matter. | **0.6** | **0.8** | -0.3 | **0.8** | 0.0 | 0.4 |
| 4. My advisor understands my academic development needs. | **0.8** | **0.9** | 0.1 | **0.9** | **-0.1** | 0.1 |
| 5. My advisor treats me with respect. | **0.6** | **0.7** | 0.4 | **0.8** | -0.2 | -0.3 |
| 6. My advisor helps me set my academic plan and to anticipate opportunities on time. | **0.8** | **0.9** | 0.0 | **0.9** | 0.0 | 0.0 |
| 7. My advisor processes my requests on time. | **0.8** | **0.9** | 0.1 | **0.9** | 0.0 | 0.0 |
| 8. I can meet with my advisor in a reasonable amount of time. | **0.8** | **0.9** | 0.1 | **0.9** | 0.1 | -0.1 |
| 9. I am satisfied with the communication methods with my advisor. | **0.8** | **0.9** | 0.1 | **0.9** | 0.0 | -0.2 |
| 10. My advisor is knowledgeable about what applies to my major. | **0.7** | **0.9** | 0.1 | **0.9** | 0.1 | -0.2 |
| 11. My advisor gives attention to my personal and social development. | **0.8** | **0.9** | -0.2 | **0.8** | 0.3 | 0.0 |

Factor loadings above .40 are in bold. F = Factor/subscale

Appendix 3

*Summary of Student Academic Advising and Counseling Questionnaire EFA for the One, Two, and Three-Factors Solutions (Cont’d)*

| SASCQ item | 1 Factor solution | 2 Factor solution | | 3 Factor solution | | |
| --- | --- | --- | --- | --- | --- | --- |
|  | F1 | F1 | F2 | F1 | F2 | F3 |
| 12. My advisor evaluates my progress in meeting my graduation requirements. | **0.8** | **0.9** | -0.1 | **0.9** | 0.1 | 0.0 |
| 13. My advisor has positively impacted my continued enrollment in the nursing program. | **0.8** | **0.9** | 0.0 | **0.9** | 0.0 | 0.1 |
| 14. My advisor is sensitive to my psychological changes. | **0.7** | **0.8** | -0.4 | **0.8** | 0.3 | 0.2 |
| 15. I prefer to contact my advisor when there is a stressful situation. | **0.6** | **0.8** | -0.2 | **0.8** | 0.0 | 0.2 |
| 16. I would recommend my advisor to other students. | **0.8** | **0.9** | 0.0 | **0.9** | 0.0 | 0.0 |
| 17. My advisor is a source of social support. | **0.8** | **0.9** | -0.1 | **0.9** | 0.0 | 0.1 |
| 18. How would you rate the overall effectiveness of your advising process? | **0.6** | **0.8** | 0.2 | **0.8** | -0.1 | -0.1 |
| SS loadings | 12.9 | 12.9 | 0.71 | 12.9 | 0.51 | 0.51 |
| Percentage of variance | 72% | 72% | 4% | 72% | 3% | 3% |
| Cumulative variance |  | 72% | 76% | 72% | 75% | 78% |
| Percentage explained |  | 95% | 5% | 93% | 4% | 4% |
| Cumulative explained |  | 95% | 100% | 93% | 96% | 100% |
| Factor correlations |  |  |  |  |  |  |
| F1 |  | 1 | 0.03 | 1 | 0.05 | 0.09 |
| F2 |  | 0.03 | 1 | 0.05 | 1 | 0.23 |
| F3 |  |  |  | 0.09 | 0.23 | 1 |

Factor loadings above .40 are in bold. SS = sum of squares. F = Factor/subscale
